# Synthesis, Structural Elucidation, and Anti-Inflammatory Activity of a Water-Soluble Derivative of Arctiin

**DOI:** 10.3390/molecules28041789

**Published:** 2023-02-14

**Authors:** Xia Xu, Xiaofeng Huang, Yuedan Zheng, Xiaoling Wang, Jing Xie, Sha Liu, Kun Guo

**Affiliations:** 1 College of Pharmacy, Southwest Minzu University, Chengdu 610200, China; 2 School of Pharmacy, Chengdu Medical College, Chengdu 610500, China; 3 The Second Affiliated Hospital of Chengdu Medical College (China National Nuclear Corporation 416 Hospital), Chengdu 610066, China

**Keywords:** arctigenin-4′-*O*-glucuronide, arctiin, water-soluble derivative, anti-inflammatory activity

## Abstract

The poor oral bioavailability of arctiin caused by its low water solubility is the biggest obstacle in developing it as a drug. In this work, a new water-soluble glucuronide derivative of arctiin (arctigenin-4′-*O*-glucuronide) was synthesized through 2,2,6,6-tetramethylpiperidine 1-oxyl mediated oxidation reaction. Subsequently, its anti-inflammatory effect was evaluated by mice acute lung injury model in vivo. The results showed that the glucuronide derivative of arctiin not only had better water solubility but also displayed improved anti-inflammatory activity in vivo, thus serving as an innovative compound in the drug development of arctiin.

## 1. Introduction

**Figure 1:**
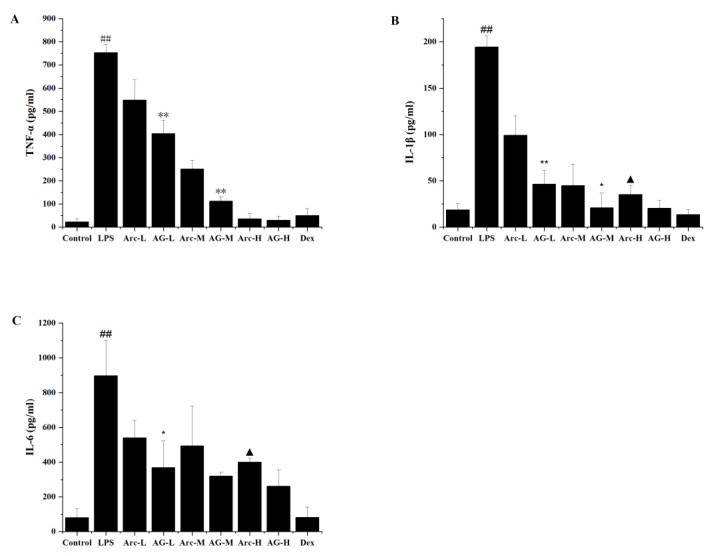
Effects of Arc and AG on cytokines of TNF-α (**A**), IL-1β (**B**), and IL-6 (**C**) in the BALF of LPS-induced ALI mice. The groups of Arc, AG, and Dex were pretreated with corresponding drugs 1 h prior to intranasal administration of LPS. The values presented are mean ± SEM of four independent experiments (*n* = 4 in each group). ^##^ *p* < 0.01 vs. Model; * *p* < 0.05, ** *p* < 0.01 vs. the same dose of Arc group; ^▲^ *p* < 0.05 vs. Dex group. Control: Control group; LPS: LPS group; Dex: LPS + Dex (5 mg/kg) group; Arc-L: LPS+Arc-low (10 mg/kg) group; Arc-M: LPS+Arc-middle (20 mg/kg) group; Arc-H: LPS+Arc-high (40 mg/kg) group; AG-L: LPS+AG-low (10 mg/kg) group; AG-M: LPS+AG-middle (20 mg/kg) group; AG-H: LPS+AG-high (40 mg/kg) group.

Fructus Arctii, the dried and mature fruit of the compositae plant *Arctium lappa* L., has a long history of use in traditional Chinese medicine. The earliest record can be traced back to 420 AD. As a crude drug included in the Chinese Pharmacopoeia, F. Arctii was a traditional Chinese medicine used to treat colds, throat irritation, mumps, measles, sores, and eczema. The main active ingredient in F. Arctii was lactone lignans. Among them, arctiin (arctigenin-4′-*O*-glucoside, Arc) (Figure 1) was considered to be the representative anti-inflammatory active ingredient of F. Arctii [1]. Its anti-inflammatory effect was not only obvious in in vitro models [2,3,4], but also showed certain efficacy in acute lung injury (ALI) [5], glomerulonephritis (GN) [6], and other inflammatory diseases [7,8,9,10]. Although Arc had shown great potential in drug development, its medicinal applicability was limited, mainly due to the poor oral bioavailability of Arc caused by the low water solubility [11], which limited the development of Arc as a drug. Therefore, improving the water solubility of Arc was key to the development of Arc as an innovative drug. The poor druggability resulting from low water solubility was a common shortcoming of natural products. Therefore, many new preparation technologies have been developed by pharmacists, such as solid dispersion [12], liposome [13], cyclodextrin inclusion [14], and nanoparticles [15]. It had been reported that the water solubility of Arc had been improved by preparing Arc self micro-emulsifying drug release system, but unfortunately, the activity had not been studied in this study [16]. In addition, the use of a large number of excipients increased the uncertainty of animal/human safety tests. Pharmaceutical chemists starting from the structure of compounds, improved the water solubility of compounds through a variety of chemical structure modification methods, such as salifying [17], introducing polar groups [18], reducing fat solubility [19], and prodrug modification, including phosphoric acid modified prodrugs [20], amino acid modified prodrugs [21], glycosylated prodrugs [22], carboxylate modified prodrugs [23], amide modified prodrugs [24], etc. Among them, glucuronization was one of the schemes to improve water solubility. As a phase II metabolite of the compound, it could not only improve the water solubility of the compound, but also completely retained the active structure of the compound. Many successful cases had emerged in the development of drugs, such as the glucuronized derivative of morphine [25], the glucuronized derivative of S-8921 [26], and the glucuronized derivative of astragaloside IV [27,28,29,30,31].

2,2,6,6-Tetramethylpiperidine 1-oxyl (TEMPO) was a classic nitroxide radical, which could electively oxidize the primary alcohol to carboxylic acid [27]. In this work, a new water-soluble glucuronide derivative of Arc, arctigenin-4′-*O*-glucuronide (AG) (Figure 1), was synthesized through TEMPO mediated oxidation reaction. This synthesis method was not only simple in operation, mild in conditions, and small in side-reactions, but also high in yield of products obtained by this method, which was suitable for large-scale production. Subsequently, the anti-inflammatory effect of AG was evaluated by the mice ALI model in vivo. The results showed that AG not only had better water solubility but also had better effective activity in vivo, which was expected to be an innovative compound for the drug development of Arc.

## 2. Results

### 2.1. Structure Elucidation of AG

Unambiguous structural assignments were made using a combination of nuclear magnetic resonance techniques in DMSO solution, including ^1^H-NMR, ^13^C-NMR, DEPT (θ = 90°, 135°), HSQC, and HMBC. The ^1^H NMR spectrum of AG showed six aromatic proton signals, three methoxyl signals, and one sugar moiety signal separately. Six proton signals upfield of δ 6.61–6.98 were observed in the ^1^H NMR spectrum, which was characteristic of two 1,3,4-trisubstituted benzene ring structures. In addition, there were three methoxyl signals at δ 3.69, 3.70, and 3.71 in the ^1^H NMR spectrum. The ^13^C NMR spectrum of AG showed two carbonyl signals, twelve aromatic carbon signals, three methoxyl carbons signals, one anomeric carbon signal, and nine saturated carbon signals, separately. Thus, the aglycone of AG could be preliminarily determined as arctigenin based on the ^1^H NMR and ^13^C data and the comparison of literature. The ^1^H and ^13^C NMR spectra displayed one anomeric signal at δ_H_ 4.88 (d, *J* = 7.56 Hz) and δ_C_ 99.87, suggesting that there was one sugar moiety in AG. Further, the carbohydrate could easily be identified as β-d-glucuronic acid by negative mode ESI-MS fragments at *m/z* 371 [M-H-176]^-^, and TLC analysis of acidic hydrolysis of AG. The attachment sites of the carbohydrates in AG were determined as C-4′ by glycosylation, which was confirmed by HMBC analysis. Under further verification by DEPT 135, DEPT 90, HSQC, and HMBC spectrums, the structure of the derivative was elucidated as arctigenin-4′-*O*-glucuronide. The ^1^H and ^13^C NMR data of AG are listed in Table 1. All spectrums used above for structure elucidation were available in Supplementary Materials.

### 2.2. Anti-Inflammatory Activity

#### 2.2.1. Effects of Arc and AG on Concentrations of IL-1β, IL-6 and TNF-α in Bronchoalveolar Lavage Fluid (BALF)

In this study, inflammatory cytokines in BALF were determined to evaluate the anti-inflammatory effects of Arc and AG. Cytokines analyzed including TNF-α, IL-1β, and IL-6 in BALF 6 h after inhalation of lipopolysaccharide (LPS) were apparently raised in LPS-challenged animals when compared to the control group. The treatment of Arc and AG groups efficiently inhibited the LPS-induced increase of the inflammatory factors, as compared with the LPS group (Figure 1). Compared with dexamethasone (Dex), AG-H performed a similar inhibitory effect on inflammatory cytokines, while Arc-H showed that the inhibitory effect on IL-6 and IL-1β was significantly lower than that of the Dex group. Furthermore, for the IL-1β and TNF-α, the low and middle doses of AG performed a more significant inhibitory effect than Arc (Figure 1A,B); For IL-6, compared with the Arc group, the low dose group of AG also showed a more significant effect on decreasing the IL-6 (Figure 1C).

#### 2.2.2. Effects of Arc and AG on Inflammatory Cell Accumulation in Lungs

**Figure 2:**
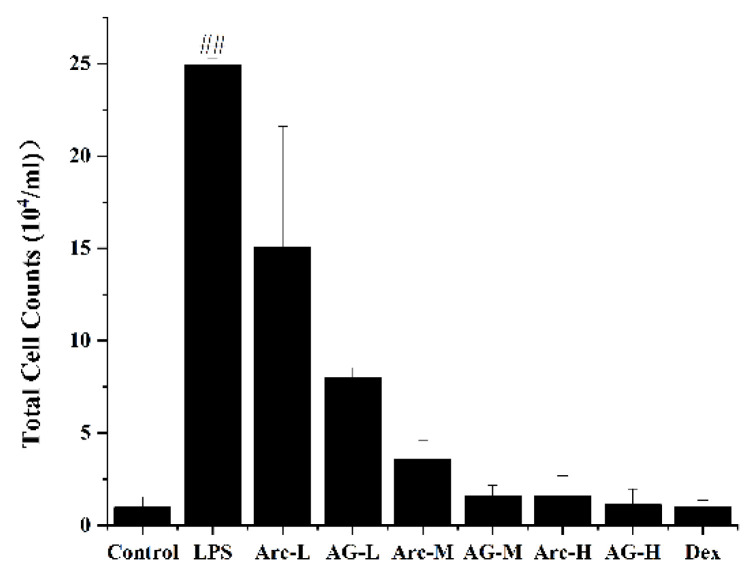
Effects of Arc and AG on inflammatory cell accumulation in lungs. The groups of Arc, AG, and Dex were pretreated with corresponding drugs 1 h prior to intranasal administration of LPS. The values presented are mean ± SEM of four independent experiments. (*n* = 3~4 in each group). ^##^ *p* < 0.01 vs. Model; Control: Control group; LPS: LPS group; Dex: LPS + Dex (5 mg/kg) group; Arc-L: LPS+Arc-low (10 mg/kg) group; Arc-M: LPS+Arc-middle (20 mg/kg) group; Arc-H: LPS+Arc-high (40 mg/kg) group; AG-L: LPS+AG-low (10 mg/kg) group; AG-M: LPS+AG-middle (20 mg/kg) group; AG-H: LPS+AG-high (40 mg/kg) group.

Inhalation of LPS will cause inflammatory cells to flow into the lungs over time. As shown in Figure 2, LPS significantly increased the total number of inflammatory cells in BALF. However, Arc and AG effectively reduced the increase in the total number of inflammatory cells induced by LPS. The high dose of Arc and AG showed similar efficacy when compared with the Dex group; Compared with the Arc group, at the same dose, the AG group had a more effective role in reducing the total number of inflammatory cells (Figure 2).

#### 2.2.3. Effects of Arc and AG on LPS-Induced Lung W/D Ratio

Pulmonary edema was one of the typical characteristics of acute lung injury. As shown in Table 2, after LPS challenged, the lung W/D Ratios of mice were increased significantly. However, the ratios could be reduced effectively by pretreating with Arc and AG. The improvement effect of a high dose of Arc and AG was similar to the Dex group. And the inhibitory effect of the AG group was better than the Arc group at a medium dose.

#### 2.2.4. Effects of Arc and AG on LPS-Mediated Lung Histopathologic Changes

**Figure 3:**
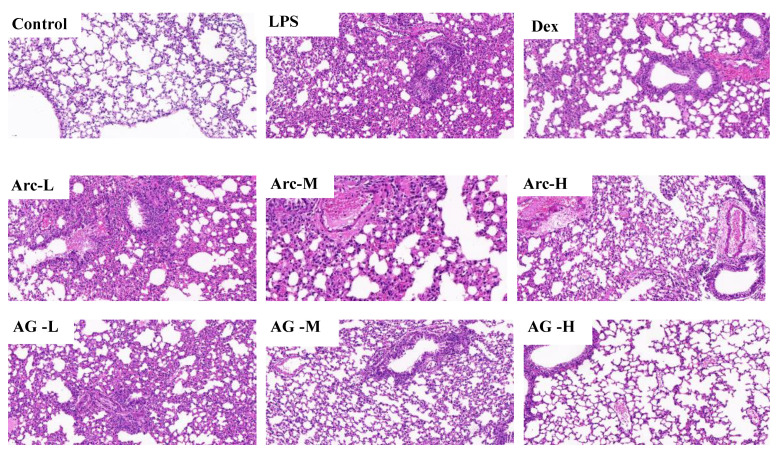
Effects of Arc and AG on histopathological changes in lung tissues in LPS-induced mice. These representative histological changes of the lung were obtained from mice of different groups (hematoxylin and eosin staining, original magnification 200×). Control: Control group; LPS: LPS group; Dex: LPS + Dex (5 mg/kg) group; Arc-L: LPS+Arc-low (10 mg/kg) group; Arc-M: LPS+Arc-middle (20 mg/kg) group; Arc-H: LPS+Arc-high (40 mg/kg) group; AG-L: LPS+AG-low (10 mg/kg) group; AG-M: LPS+AG-middle (20 mg/kg) group; AG-H: LPS+AG-high (40 mg/kg) group.

Compared to the control group, lung specimens from the LPS group demonstrated obvious histopathologic abnormalities, which included the severe hemorrhage in the alveolus, infiltration of inflammatory cells into the interstitial and alveolar spaces, interstitial edema, and widespread alveolar wall thickness (Figure 3). With regard to the treatment groups (Arc, AG, and Dex), the severity of histopathologic changes in lung tissues was slimmer than that in the LPS group, especially on inflammatory cell infiltration and hemorrhage. In addition, compared with the Arc group, the AG group showed a better improvement effect at the same dose.

Table 3 showed the average score of each category, and the total score was calculated by adding the individual scores of each category. The average score of each group showed that there was no lesion in the control group, and the lesion in the LPS group was the most serious. In addition, the drug pretreatment of Arc, AG, and Dex could effectively inhibit the pathological changes in lung tissue. According to the Damage degree: 2 > 3 > 4 > 5 > 6 = 7 > 8, the high dose of AG performed the most significant inhibitory action. Both Arc and AG could effectively prevent LPS-induced lung injury, but the AG group showed better Inhibitory action, especially in the treatment of Interstitial edema and hemorrhage, and a high dose of AG even performed better effect than dexamethasone.

#### 2.2.5. Effects of Arc and AG on LPS-Induced Myeloperoxidase (MPO) Activity

**Figure 4:**
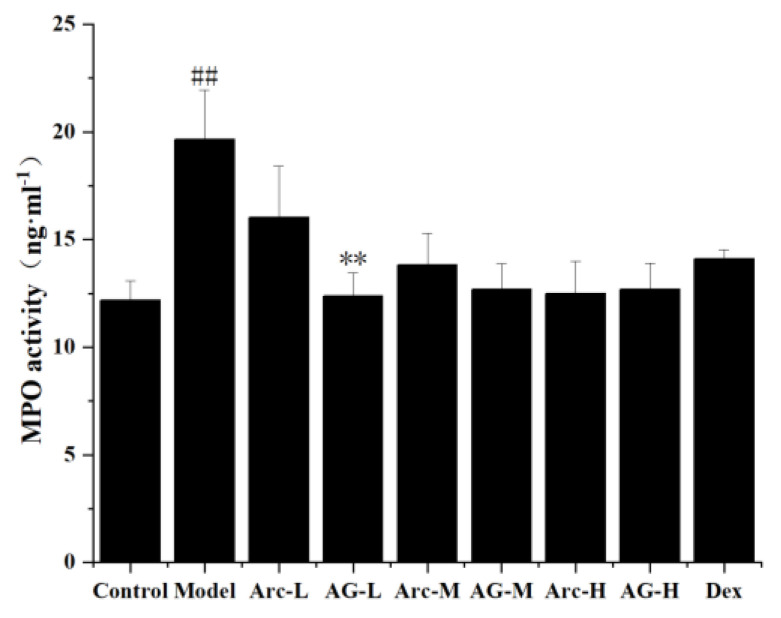
Effects of Arc and AG on MPO activity in lung tissues of LPS-challenged mice. The groups of Arc, AG, and Dex were pretreated with corresponding drugs 1 h prior to intranasal administration of LPS. The values presented are mean ± SEM of four independent experiments. (*n* = 3~4 in each group). ^##^ *p* < 0.01 vs. Model; ** *p* < 0.01 vs. the same dose of Arc group; Control: Control group; LPS: LPS group; Dex: LPS + Dex (5 mg/kg) group; Arc-L: LPS+Arc-low (10 mg/kg) group; Arc-M: LPS+Arc-middle (20 mg/kg) group; Arc-H: LPS+Arc-high (40 mg/kg) group; AG-L: LPS+AG-low (10 mg/kg) group; AG-M: LPS+AG-middle (20 mg/kg) group; AG-H: LPS+AG-high (40 mg/kg) group.

As an important index to evaluate the aggregation of inflammatory cells in lung tissue, this study measured the activity of MPO. Compared with the control group, the concentration of MPO in lung tissue was significantly increased after LPS administration. However, the administration of Arc and AG one hour before the LPS challenge significantly inhibited this change. In addition, the inhibitory effect of AG was more significant at low doses (Figure 4).

## 3. Materials and Methods

### 3.1. Apparatus and Materials

Arctiin was purchased from Lemeitian (Chengdu, China). Potassium bromide for IR was spectral grade, purchased from Kermel (Tianjin, China). All chemicals used for synthesis were of analytical grade. D101 macroporous adsorption resin purchased from Tianjin Guangfu Fine Chemical Research Institute (Tianjin, China). AG was synthesized from Arctiin with structure characterization by multiple spectroscopic analysis (IR, HR-ESI-MS, ^1^H-NMR, ^13^C-NMR, DEPT (θ = 90°, 135°), HSQC, and HMBC). Lipopolysaccharide (LPS) was purchased from Sigma-Aldrich. Mouse TNF-α, IL-6, IL-1β, and MPO ELISA kits were purchased from MULTI SCIENCES (Hangzhou, China).

### 3.2. Preparation of AG

**Scheme 1:**
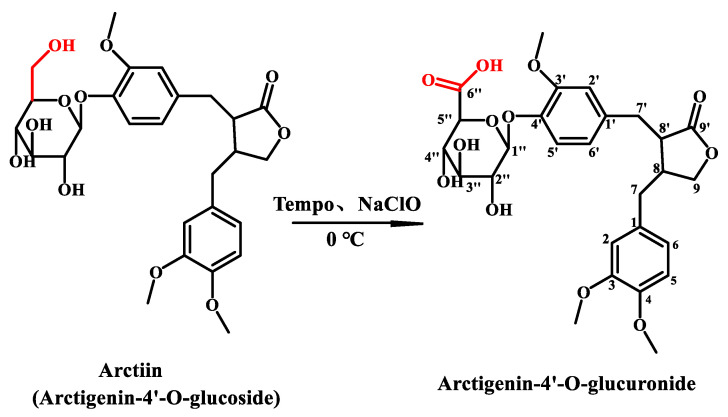
Structures of Arc and AG.

400 mg Arc was added to 50 mL 60% THF (by volume) solution containing 160 mg Na_2_CO_3_ (Sinopharm Chemical Reagent Co., Ltd., Shanghai, China), 60 mg NaHCO_3_ (Sinopharm Chemical Reagent Co., Ltd., Shanghai, China), and 40 mg TEMPO (Sinopharm Chemical Reagent Co., Ltd., Shanghai, China). The reaction mixture was cooled to about 0 °C in ice water, then NaClO was added dropwise until the thin-layer chromatography (TLC) monitored that the starting material was completely reacted, and finally 1.5 mL methanol was added to terminate the reaction. The reaction solution was concentrated under reduced pressure to remove the THF. The residue was purified with D101 macroporous resin using water and 40% ethanol as eluent, respectively. Collect 40% ethanol eluent and removed the solvent to dryness under vacuum to obtain 353 mg of product with a yield of 86%. The reaction was shown in Scheme 1.

AG was obtained as a white powder with [α${]}_{20}^{D}$ − 48° (c, 1.0, CHCl_3_), and IR (KBr) γ_max_ 3419, 2928, 1771, 1609, 1521, 1429, 1271, 1378, 1158, 1075 and 1026 cm^−1^. Its molecular formula was established as C_27_H_32_O_12_ by HR-ESI-MS (*m/z* 547.1883 [M-H]^–^, calcd. for C_27_H_31_O_12_: 547.5359), requiring 12° of unsaturation. The ESI-MS^2^ fragments were *m/z* 371 [M-H-glcA]^–^ and 357 [M-glcA-CH_3_]^–^. The TLC Rf value was 0.5 (CHCl_3_-MeOH-H_2_O-CH_3_COOH, 2:1:0.025:0.075). The solubility of AG was 227 mg/mL in pure water at 25 °C.

### 3.3. Evaluation of Anti-Inflammatory Activity

#### 3.3.1. Animals and Experimental Design

Five-week-old male Kunming mice (18–22 g) were purchased from Chengdu Dashuo Experimental Animal Co., Ltd. (Chengdu, China) and raised at a controlled temperature of 22.0 ± 2 °C and relative humidity of 50–60% on a 12 h light/12 h dark cycle with free access to food and drink. The animal experiments were conducted in accordance with the protocol of the National Act on the Use of Experimental Animals (China) and were approved by the Sichuan Committee on Laboratory Animals (approval number SYXK2014−187).

After one week of adaptation to general meals, the mice were divided into the following nine groups randomly (*n* = 12 mice per group). The groups included the control group, LPS groups, Arc high-dose (Arc-H, 40 mg/kg) group, Arc middle-dose (Arc-M, 20 mg/kg) group, Arc low-dose (Arc-L, 10 mg/kg) group, AG high-dose (AG-H, 40 mg/kg) group, AG middle-dose (AG-M, 20 mg/kg) group, AG low-dose (AG-L, 10 mg/kg) group, and Dexamethasone (Dex, 5 mg/kg) group.

The groups of Arc, AG, and Dex were given corresponding drug pretreatment by gavage 1 h prior to intranasal administration of LPS, while the control group and LPS group were treated intranasally with 50 μL of PBS. After 1 h, all mice except the blank control group were given LPS by intranasal administration. Six hours after LPS stimulation, the mice were euthanized. After that, BALF and lung tissue samples were harvested.

#### 3.3.2. Collection of BALF and Determination of TNF-α, IL-1β, and IL-6 Levels

After the mice were euthanized, the skin of the neck was cut open with scissors, and the neck muscles and subcutaneous tissues were separated longitudinally with curved tweezers so that the trachea could be fully exposed. Then, the trachea was cannulated and 0.5 mL PBS was injected into the lungs of mice. After careful flushing, draw out the liquid and put it into a centrifuge tube. Repeat the flushing process three times to obtain the BALF 1.2–1.5 mL (recovery rate is more than 80%).

The obtained BALF was centrifuged (4 °C, 3000 r/min, 10 min), and the supernatant was used for the subsequent determination of inflammatory factors, while the bottom cells were collected for the counting of inflammatory cells. The level of TNF-α, IL-1β, and IL-6 in BALF were determined by an enzyme-linked immunosorbent assay (ELISA) kit. All procedures are in accordance with the manufacturer’s instructions.

#### 3.3.3. Cell Counting

The bottom cells obtained by BALF centrifugation were rinsed twice with 0.5 mL hank’s solution (without Ca^2+^, Mg^2+^) under the same conditions (4 °C, 3000 r/min) for 5 min each time. The washed cell sediment was mixed with 0.5 ml of normal saline for cell counting.

#### 3.3.4. Lung Wet-to-Dry Weight (W/D) Ratio

The mice without BALF obtained were dissected and the right lungs of mice were excised, dried with filter paper, weighed to obtain “wet” weight (W), and then placed in an oven at 80 °C for 72 h to obtain “dry” weight (D). After that, dried it for 5 h at 80 °C, weighed it three times, and checked whether it is weighed. The ratio of the wet lung to the dry lung (W/D) was calculated to assess tissue edema.

#### 3.3.5. Histopathologic Evaluation of the Lung Tissue

Histopathological examination was performed on mice without BALF collection. The left lung tissue samples of mice were fixed in 10% neutral buffered formalin for 48 h, then dehydrated in ascending series ethanol, including 70%, 80%, 90%, 95%, and 100% ethanol, transparent in xylene, soaked in melted paraffin, and finally embedded in paraffin and sliced. embedded in paraffin and sliced. After hematoxylin-eosin (H&E) staining (Follow the instructions), the pathological changes of lung tissue were observed under the microscope.

According to the pathological injury characteristics of each sample, the histological score was carried out. The severity of lung injury ranges from 0 to 4 (the “0” meant normal while the “4” represented severely) and falls into the following categories: neutrophil infiltration, interstitial edema, hemorrhage, and congestion. The total score was calculated by adding the individual scores of each category.

#### 3.3.6. Pulmonary MPO Activity Assay

The right lung of the mice was rinsed with precooled PBS to remove residual blood and inhaled dry on the filter paper. After weighing, the tissue was cut into pieces with scissors. Then the cut tissue was added to PBS in a 1:9 weight volume ratio. The cut tissue was put into a low-temperature homogenizer to make homogenate. Finally, the homogenate was centrifuged at 4 °C at 5000 r/min for 10 min. The supernatant was taken and tested according to MPO Elisa kit.

#### 3.3.7. Statistical Analysis

IBM SPSS Statistics (R26.0.0.0, IBM, Chicago, IL, USA) was used for the statistical analysis of data through one-way ANOVA. The experimental data were expressed as mean ± standard deviation (x ± s); Comparing the difference between groups, *p* < 0.05 indicates a statistical difference.

## 4. Discussion

The mouse model of acute lung injury was established to verify the biological activity of AG. ALI may be induced by many factors, such as foreign body inhalation, pathogenic bacteria infection (including novel coronavirus), primary injury, etc. LPS was the main component of the outer membrane of gram-negative bacteria cells, which could stimulate the recruitment and activation of inflammatory cells in the lungs, lead to systemic inflammatory reaction and immune activation, and produce tissue damage [32]. In this experiment, the ALI model was established by intranasal administration of LPS. The pathological changes in the lung tissue of the model group mice showed alveolar hemorrhage, destruction of alveolar tissue, thickening of alveolar septa, and infiltration of inflammatory cells. It could be seen that the model was successfully established, and it was further confirmed by the increase of W/D in lung tissue, the number of inflammatory cells accumulated in the lung, the activity of MPO, and the significant increase in inflammatory factor production.

Hormone therapy was the main method for drug treatment of acute lung injury. Glucocorticoids could effectively reduce the inflammatory reaction, but had no tissue repair effect. Long-term use of hormone drugs would affect the body’s organs and even lead to systemic diseases [33,34]. Therefore, a growing number of researchers turned their attention to traditional Chinese medicine, hoping to find potential drugs for treating ALI.

It had been reported that Arc had a certain inhibitory effect on LPS-induced acute lung injury [5], but the oral bioavailability of Arc was low because of its poor solubility. In this study, the water-soluble derivative of Arc, AG, obtained by tempo-mediated oxidation method, effectively improved the inflammatory cell infiltration, pulmonary edema, pulmonary hemorrhage, interstitial edema, and other symptoms of the model group, effectively inhibited the increase of inflammatory factors in BALF of mice, and compared with the same dose of Arc, the improvement effect and inhibition effect were enhanced. Therefore, the AG obtained by tempo-mediated oxidation did indeed improve their bioavailability by improving the solubility of Arc as expected.

Our research showed that AG was beneficial to ALI. And in mice with ALI induced, AG was more effective than Arc in inhibiting the release of inflammatory factors in the lungs of ALI mice, reducing the inflammatory response, reducing pulmonary edema, and improving lung injury.

## Figures and Tables

**Table 1:** ^1^H-NMR (600 MHz) and ^13^C-NMR data (150 MHz) of AG in DMSO (δ in ppm).

Position	δ_H_	δ_C_	DEPT	Position	δ_H_	δ_C_	DEPT
1		131.68	C	6′	6.84 (7.6, d)	120.4	CH
2	6.76 (s)	112.46	CH	7′	2.45 (m)	36.83	CH_2_
3		148.64	C	8′	2.59 (m)	40.62	CH
4		145.31	C	9′		178.4	C
5	6.65 (8.2, 2.0, dd)	115.17	CH	Glu-1″	4.88 (7.6, d)	99.87	CH
6	6.97 (8.3, d)	121.28	CH	Glu-2″	3.20–3.28 (m)	76.68	CH
7	2.80 (m)	33.39	CH_2_	Glu-3″	3.20–3.28 (m)	73.08	CH
8	2.73 (m)	45.61	CH	Glu-4″	3.20–3.28 (m)	74.29	CH
9	3.86 (8.8, t), 4.09 (8.2, t)	70.68	CH_2_	Glu-5″	3.52 (m)	71.99	CH
1′		131.19	C	Glu-6″		173.1	C
2′	6.68 (s)	111.91	CH	OCH_3_	3.71 (s)	55.68	CH_3_
3′		148.69	C	OCH_3_	3.70 (s)	55.51	
4′		147.34	C	OCH_3_	3.69 (s)	55.45	
5′	6.62 (8.4, 2.0, dd)	113.94	CH				

**Table 2:** Effects of Arc and AG on pulmonary edema.

Groups	Dose/(mg·kg^−1^)	Lung W/D/(%)
Control	-	3.92 ± 0.06
LPS	-	5.02 ± 0.08 ^##^
Arc-L	10	4.84 ± 0.38
AG-L	10	4.73 ± 0.28
Arc-M	20	4.51 ± 0.17
AG-M	20	4.47 ± 0.5 *
Arc-H	40	4.32 ± 0.07
AG-H	50	4.29 ± 0.07
Dex	5	4.2 ± 0.12

The values presented are mean ± SEM of four independent experiments. (*n* = 3~4 in each group). The groups of Arc, AG, and Dex were pretreated with corresponding drugs 1 h prior to intranasal administration of LPS. ^##^ *p* < 0.01 vs. Model; * *p* < 0.05 vs. the same dose of Arc group. Control: Control group; LPS: LPS group; Dex: LPS + Dex (5 mg/kg) group; Arc-L: LPS+Arc-low (10 mg/kg) group; Arc-M: LPS+Arc-middle (20 mg/kg) group; Arc-H: LPS+Arc-high (40 mg/kg) group; AG-L: LPS+AG-low (10 mg/kg) group; AG-M: LPS+AG-middle (20 mg/kg) group; AG-H: LPS+AG-high (40 mg/kg) group.

**Table 3:** Effects of Arc and AG on LPS-induced lung morphology. Lung Histology Score.

Groups	Neutrophil Infiltration	Interstitial Edema	Hemorrhage	Congestion	Scores
Control	0	0	0	0	0
LPS	3	3	4	3	13
Arc-L	2	2	2.5	3	9.5
AG-L	2.5	2	2.5	2	9
Arc-M	2	1	2	2	7
AG-M	2.5	0	2	2	6.5
Arc-H	1	1.5	2	2	6.5
AG-H	0.5	0	0	1	1.5
Dex	1	1	2	1	5

Lung injury was graded from 0 (normal) to 4 (severe) in four categories: Neutrophil infiltration, Interstitial edema, Hemorrhage, and Congestion. The total lung injury score was calculated by adding up the individual scores of each category. The values presented are mean ± SEM of four independent experiments. (*n* = 4 in each group). Control: Control group; LPS: LPS group; Dex: LPS + Dex (5 mg/kg) group; Arc-L: LPS+Arc-low (10 mg/kg) group; Arc-M: LPS+Arc-middle (20 mg/kg) group; Arc-H: LPS+Arc-high (40 mg/kg) group; AG-L: LPS+AG-low (10 mg/kg) group; AG-M: LPS+AG-middle (20 mg/kg) group; AG-H: LPS+AG-high (40 mg/kg) group.

## Data Availability

Not applicable.

## Supplementary Materials

The following supporting information can be downloaded at: https://www.mdpi.com/article/10.3390/molecules28041789/s1, Figure S1: HR-ESI-MS spectra; Figure S2: ESI-MS^2^ spectra; Figure S3: ^1^H-NMR spectra; Figure S4: ^13^C-NMR spectra; Figure S5: DEPT (θ = 90°), Figure S6: DEPT (θ = 135°) spectra; Figure S7: HMBC spectra and Figure S8: HSQC spectra.

## Sample Availability

Samples of arctigenin-4′-*O*-glucuronide is available from the authors.
