# Supplementary material for: Synthesis, Structural Elucidation, and Anti-Inflammatory Activity of a Water-Soluble Derivative of Arctiin
Source: Molecules. 2023 Feb 14;28(4):1789. doi: 10.3390/molecules28041789 (PMC9961579; doi:10.3390/molecules28041789)
Supplement: Supplementary file 1 [file molecules-28-01789-s001.zip › molecules-2164089-supplementary.pdf]

Supplementary data

Supplementary data (HR-ESI-MS, ESI-MS^2, ^{1}H-NMR, ^{13}C-NMR, DEPT (θ = 90°), DEPT (θ = 135°), HMBC and HSQC spectra for arctigenin-4'-O-glucuronide) associated with this article can be found as following:

Figure S1. HR-ESI-MS

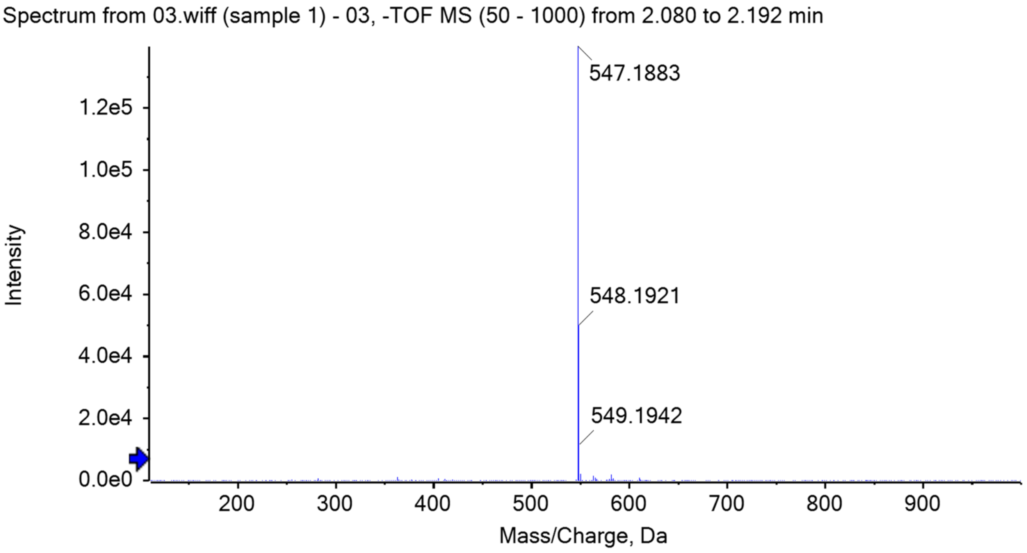

Figure S2. ESI-MS^2

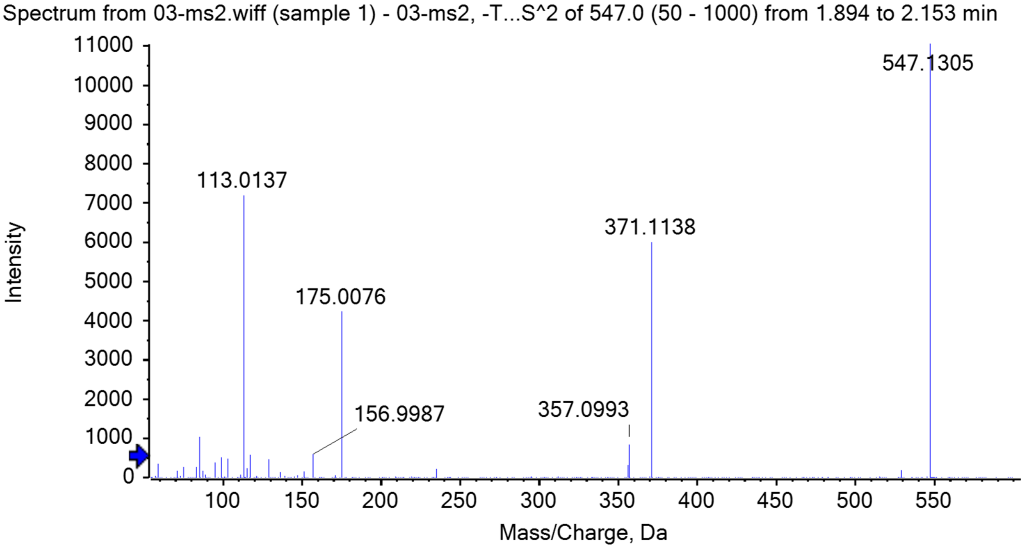

Figure S3. ^{1}H-NMR (DMSO-d_6)

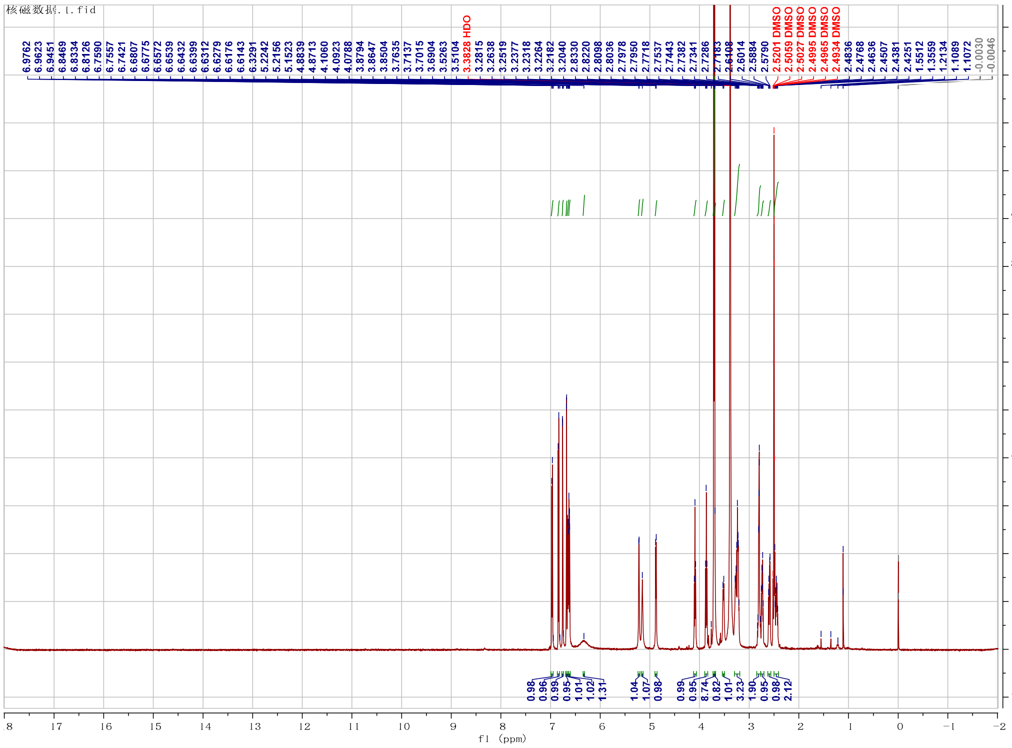

Figure S4. ^{13}C-NMR (DMSO-d_6)

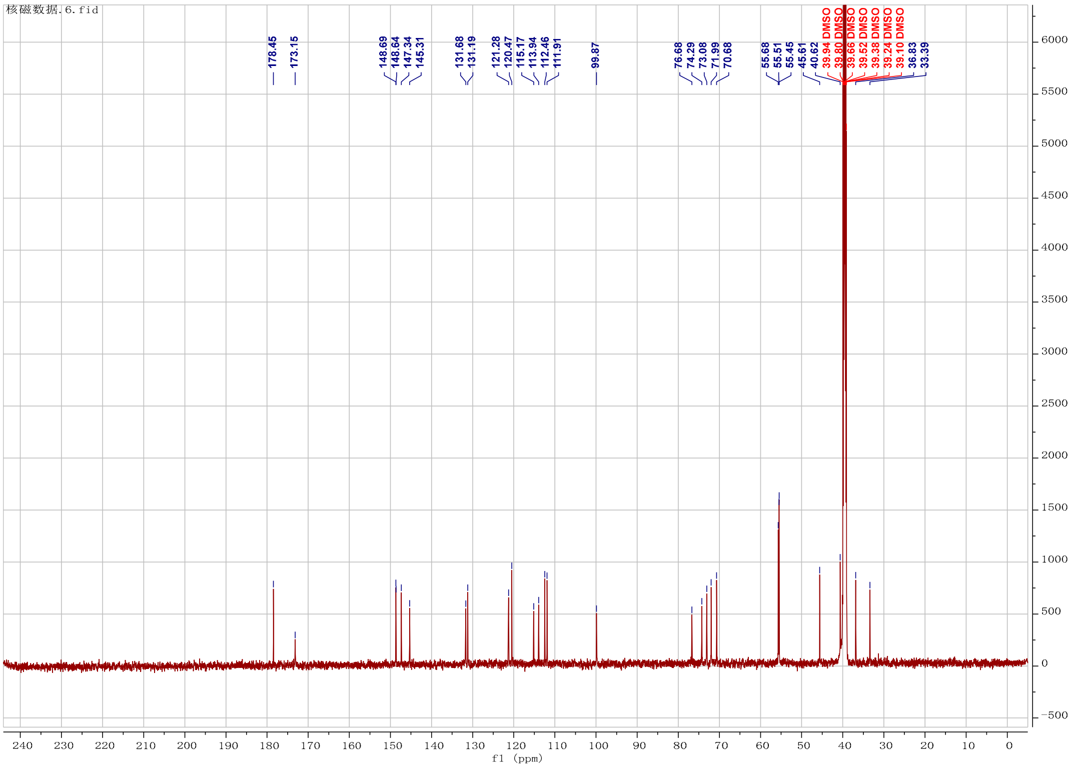

Figure S5. DEPT (θ = 90°) (DMSO-d_6)

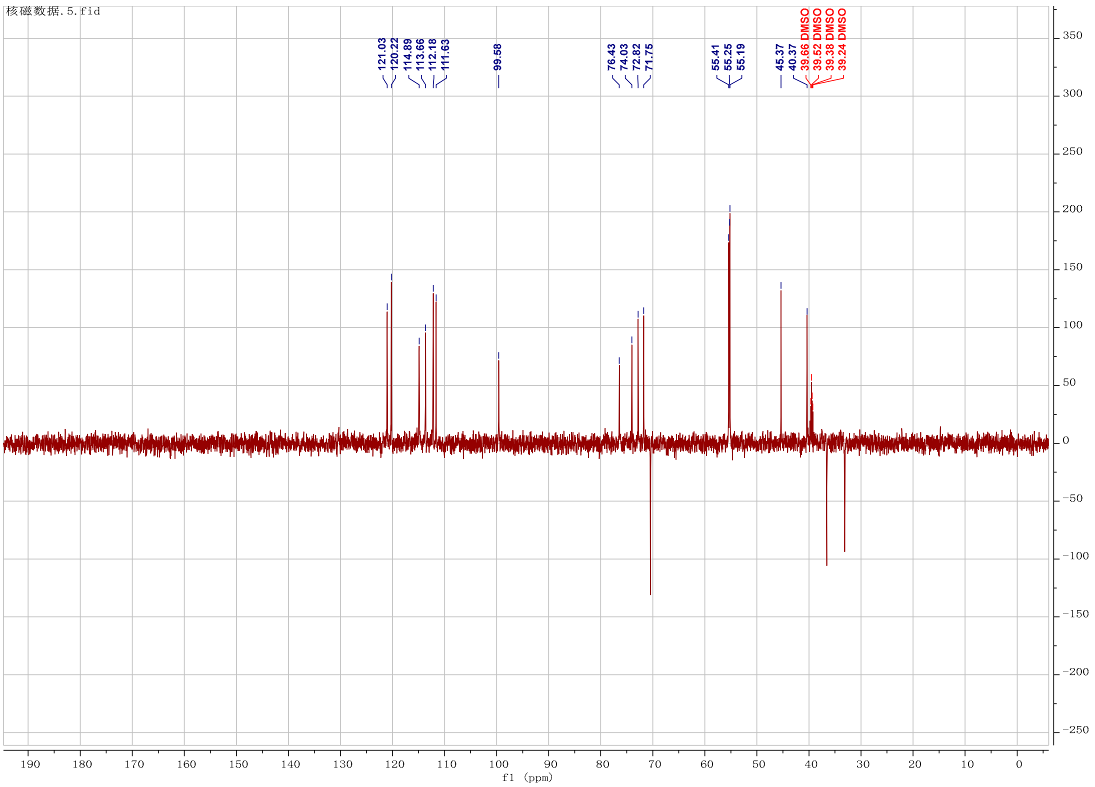

Figure S6. DEPT (θ = 135°) (DMSO-d_6)

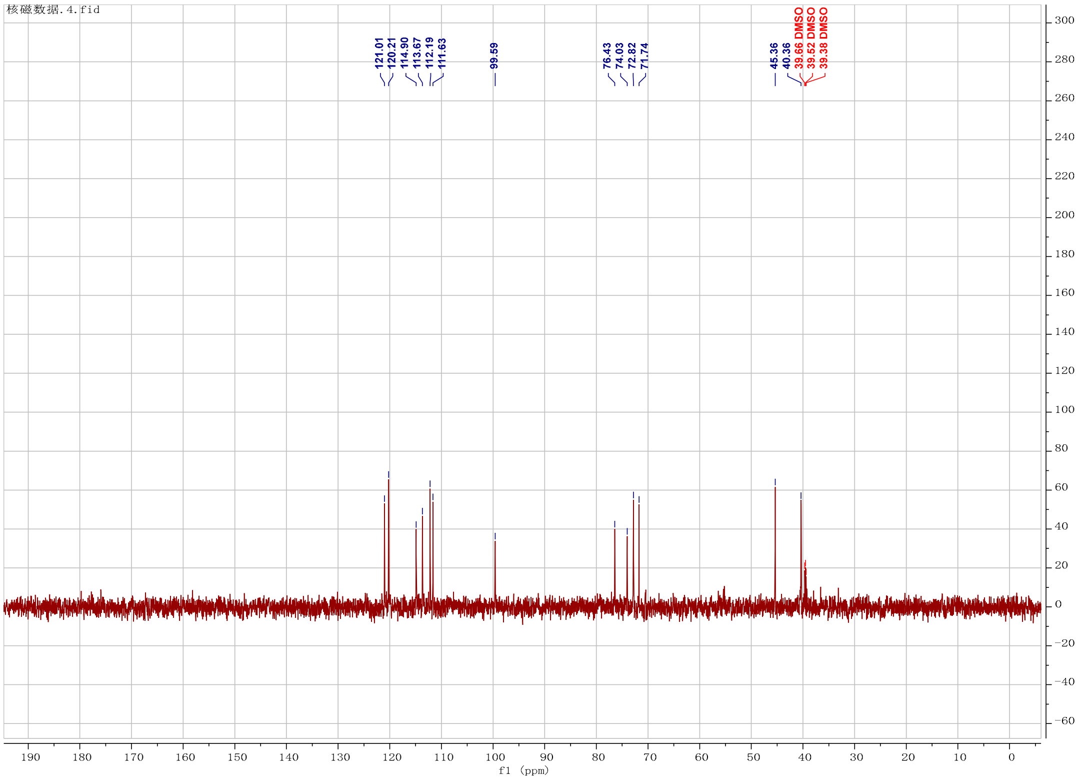

Figure S7. HMBC (DMSO-d_6)

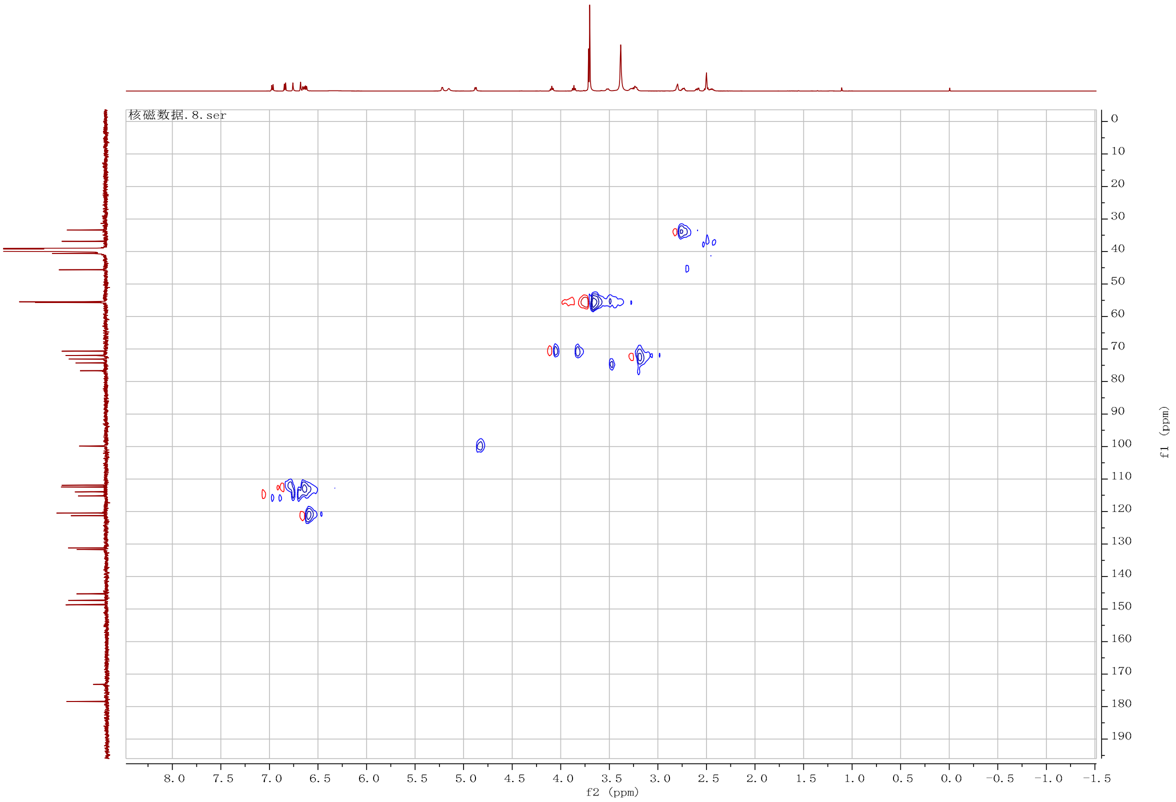

Figure S8. HSQC (DMSO-d_6)

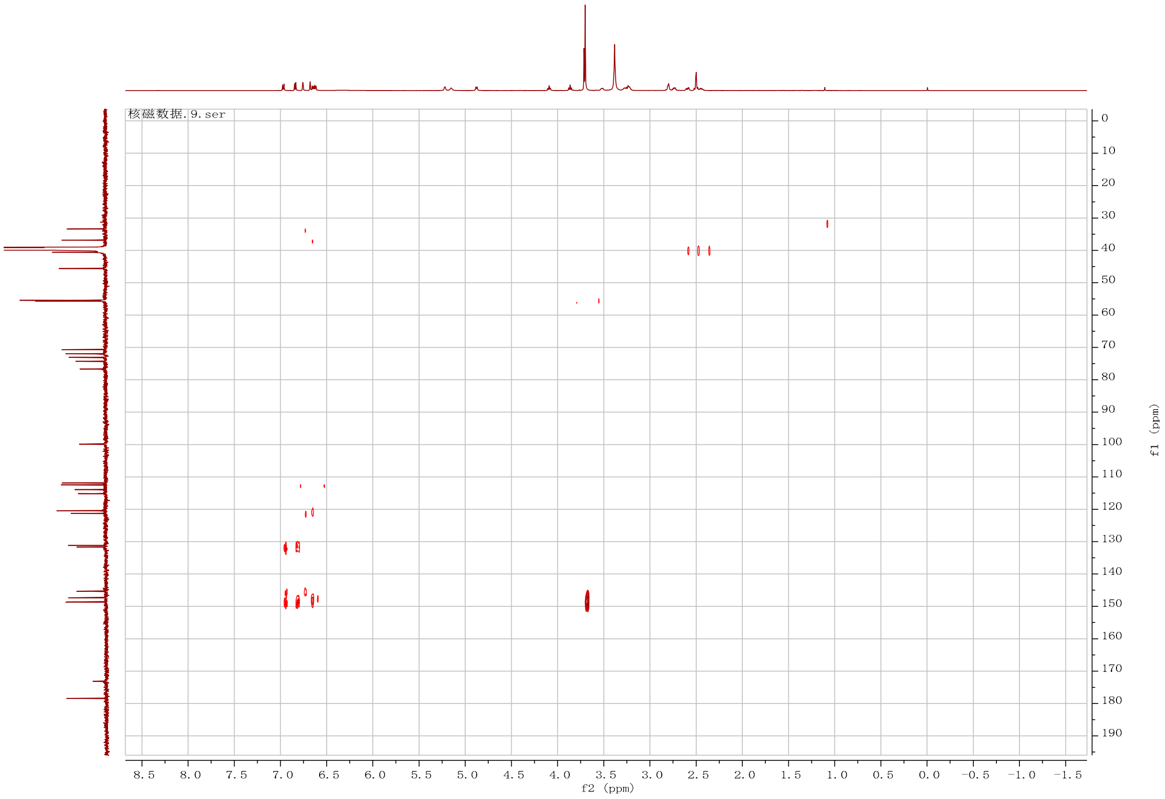

IR (KBr)

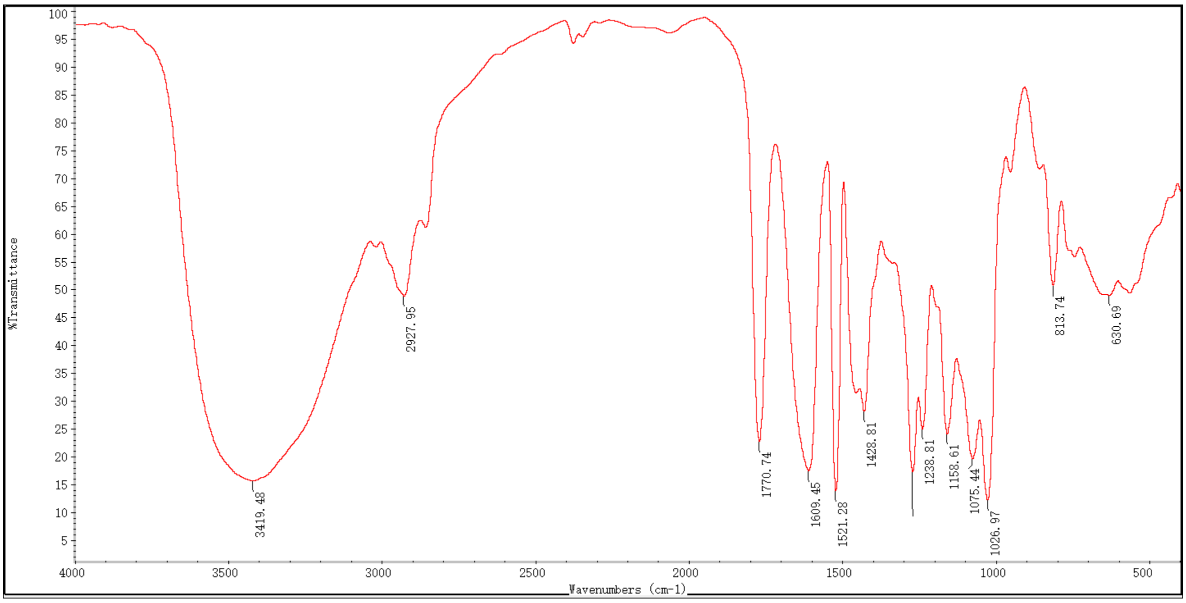
%Transmittance
Wavenumbers (cm-1)
3419.48
2927.95
1770.74
1609.45
1521.28
1428.81
1238.81
1158.61
1075.44
1026.97
813.74
630.69
